# A Simple Ratio in a Complex Disease: Exploring the Neutrophil-to-Lymphocyte Ratio in Idiopathic Pulmonary Fibrosis

**DOI:** 10.3390/jcm14145100

**Published:** 2025-07-18

**Authors:** Giorgio Monteleone, Luca Passantino, Jacopo Simonetti, Bruno Iovene, Francesco Varone, Paolo Cameli, Giacomo Sgalla, Luca Richeldi

**Affiliations:** 1 Department of Cardiovascular and Pulmonary Sciences, Catholic University of Sacred Heart, 00168 Rome, Italy; giorgio.monteleone1995@gmail.com (G.M.); passantino.luca01@gmail.com (L.P.); jacopo.simonetti@guest.policlinicogemelli.it (J.S.); luca.richeldi@policlinicogemelli.it (L.R.); 2 Fondazione Policlinico Universitario Agostino Gemelli IRCCS, 00168 Rome, Italy; bruno.iovene@policlinicogemelli.it (B.I.); francesco.varone@policlinicogemelli.it (F.V.); 3 Respiratory Diseases Unit, Department of Medicine, Surgery and Neurosciences, University of Siena, 53100 Siena, Italy; paolo.cameli@unisi.it

**Keywords:** neutrophil-to-lymphocyte ratio, idiopathic pulmonary fibrosis, inflammation, biomarker

## Abstract

The neutrophil-to-lymphocyte ratio (NLR) is a simple, inexpensive and easily accessible inflammatory biomarker that reflects the balance between innate and adaptive immunity. In recent years, NLR has emerged as a potential prognostic and disease severity marker for different diseases, including idiopathic pulmonary fibrosis (IPF), a progressive and fatal interstitial lung disease with a highly variable course and poor prognosis. Several studies have highlighted that NLR can be associated with several clinical outcomes such as lung function decline, increased risk of hospitalization, acute exacerbation of IPF, and mortality over time. It might also correlate with overall survival in the course of antifibrotic therapy and validated prognostic score as a gender–age–physiology score. Despite these findings, the clinical use of NLR remains limited due to its non-specific nature, the lack of standardized cut-off values, and high variability related to demographic factors, comorbidities and medications. Hence, NLR may display the underlying immune dysregulation in IPF and could be exploited as a non-invasive tool for risk stratification and disease monitoring. Further studies are needed to confirm and validate its use in IPF and to establish reliable cut-off values in clinical applications.

## 1. Introduction

The identification of reliable biomarkers is crucial for monitoring the unpredictable course of idiopathic pulmonary fibrosis (IPF), which can range from a slow decline in lung function over time to a rapid progression or acute exacerbation, which is linked to a markedly high risk of mortality [1]. Biomarkers are measurable biological indicators that can be used to forecast, monitor, or assess clinical outcomes and endpoints [2]. They can support disease screening, diagnosis, staging, follow-up, prognosis, assessment of treatment response, and identification of cell types [2]. Within serum biomarkers, the neutrophil-to-lymphocyte ratio (NLR) is an inflammatory marker calculated based on circulating neutrophil-to-lymphocytes [3]. Although standardized cut-off values have not been established, NLR has gained attention as diagnostic, prognostic and disease severity marker across a broad spectrum of disease, including cardiovascular, rheumatic, gastrointestinal, and pulmonary diseases such as interstitial lung diseases (ILDs) [3,4,5]. Amid ILDs, IPF is the most common and prevalent fibrosing interstitial lung disease (f-ILD), whose hallmark is the presence of a radiological or histological pattern of usual interstitial pneumonia (UIP) on chest high-resolution computed tomography or lung tissue specimens, respectively [6]. It is characterized by an ongoing fibrotic degeneration of the lung interstitium that leads to progressive pulmonary function impairment, ultimately resulting in respiratory failure and death [7]. Although antifibrotic drugs such as pirfenidone and nintedanib have improved the prognosis of patients by slowing down disease progression, the management of IPF remains challenging due to its high mortality and lack of biomarkers that can be used to predict clinical outcomes and inform the therapeutic response [8]. Several studies highlighted that NLR can play a potential role in IPF management. In particular, higher values and a progressive increase over time may be associated with lung function decline, worsening respiratory symptoms, increased risk of hospitalization and mortality in IPF patients [9,10]. Additionally, the NLR has been assessed in patients with acute exacerbation of IPF (AE-IPF) suggesting its potential for predicting survival outcome [11]. Our review aims to provide a comprehensive overview of the current findings on the role of NLR as a biomarker in IPF, including its future perspective.

## 2. Methods

**Figure 1:**
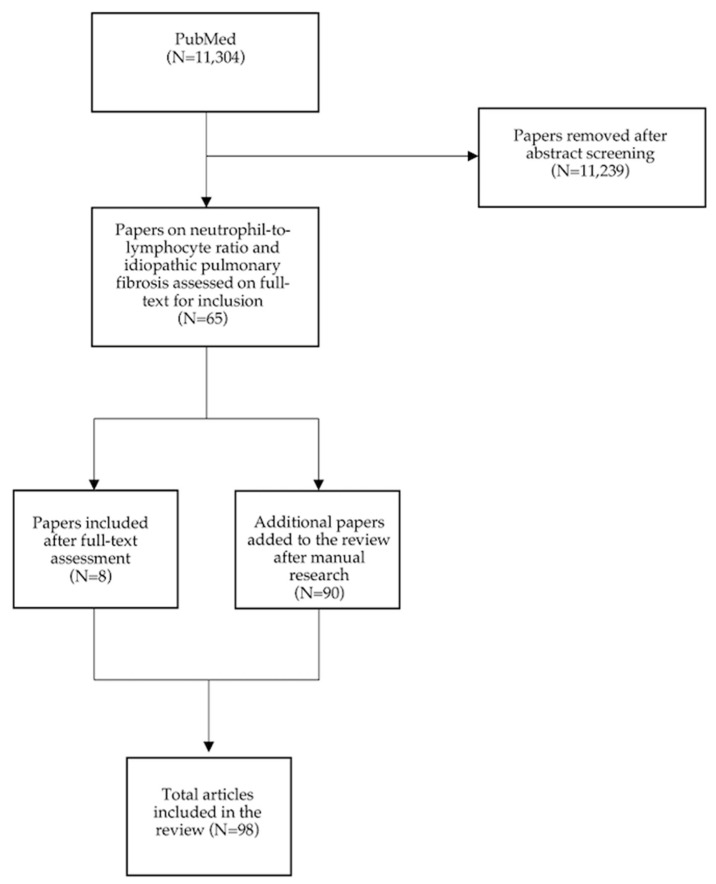
**Flow diagram of the literature search and selection process.** A total of 11,304 records were initially retrieved from the PubMed database. Among them, 11,239 were excluded after an assessment of their titles and abstracts. Sixty-five articles pertaining to the neutrophil-to-lymphocyte ratio and idiopathic pulmonary fibrosis were selected for full-text assessment. Of these, only 8 papers were included in the review. The other 90 additional articles were identified through manual searches and subsequently added to the manuscript.

The literature search for this narrative review was conducted via the PubMed database, using a combination of keywords such as “neutrophil-to-lymphocytes ratio”, “NLR”, “idiopathic pulmonary fibrosis”, “IPF”, “interstitial lung diseases”, “ILD”, and “biomarkers” to identify relevant studies. Only articles published in English were considered for data extraction. Additional references were added by manually screening the bibliography of the key articles. Finally, the selected studies were critically reviewed by the authors and included in the reference list (Figure 1).

## 3. Pathogenesis of Idiopathic Pulmonary Fibrosis

The pathogenesis of IPF is multifactorial and has been determined by multiple mechanisms and signaling pathways that have yet to be fully elucidated. Recent findings suggested that complex mechanisms such as epigenetics, cellular senescence, aging, aberrant recapitulation of developmental pathways, immune system cells dysregulation, proteostasis, and mitochondrial dysfunction can contribute to IPF pathogenesis [12,13,14,15,16]. Nevertheless, the interaction between genetic predisposition (gene polymorphisms and rare mutations) and environmental factors like active and passive smoking, microbial infections, inhaled antigens, occupational exposure, and pollutants still remains the main pathogenetic hypothesis [12,13,14,17].

Environmental factors can prime a wide range of epigenetic modifications by triggering changes in gene expression without altering the DNA sequence [18]. These changes are induced by non-coding microRNA (miRNA), histone changes and DNA hypo/hyper-methylation processes [12,14]. MiRNAs are short and non-coding RNA sequences that can modify protein synthesis by interfering with post-transcriptional mechanisms [19]. Amid these, the increased expression of miR-154, which inhibits Wnt/beta-catenin profibrogenic pathway suppressors, and the reduced expression of miR-30a, miR-30d and miR-92a appeared to target developmental pathways and promote fibrosis development [13].

Cellular senescence contributes to aging through a pro-inflammatory secretory pattern and inhibition of cell cycle and apoptosis [13,20]. The concurrent secretory pattern, mediated by interleukin-1-beta, alongside the inhibition of myofibroblasts’ apoptosis triggers epithelial–myofibroblast transition (EMT), thereby driving fibrosis progression [13,20]. Therefore, epigenetics mechanisms, cellular senescence and changes in telomeres length facilitate both aging and aberrant recapitulation of developmental pathways, resulting in IPF onset and development [13,20,21].

Additionally, endoplasmic reticulum dysfunction, impaired proteostasis, and reduced autophagy are additional aging-related mechanisms which, in turn, trigger transforming growth factor-beta (TGF-β) signaling and impair mitochondrial homeostasis in type-II alveolar epithelial cells (AECIIs) [15,22]. These changes, through reduced expression of PTEN-induced putative kinase 1, a mitochondrial regulatory kinase protein, appear to contribute to EMT and myofibroblasts differentiation leading to fibrosis [22,23].

Imbalances in both innate and adaptive immune response, involving cells like neutrophils, mast cells, macrophages, monocytes, lymphocytes and immune lymphoid cells (ILCs), have been linked to fibrogenesis [14,17,24]. While neutrophils are responsible for extracellular matrix (ECM) remodeling and fibroblasts’ differentiation via the release of neutrophils elastase (NE) and neutrophils extracellular traps (NETs), mast cells can perpetrate inflammation by releasing interleukin-4 (IL-4), interleukin-10 (IL-10) and interleukin-13 (IL-13) and tissue remodeling via TGF-β secretion [14,25].

The role of monocytes, which are circulating progenitors of macrophages, in IPF pathogenesis is still debated. Monocytes may participate in fibrogenesis through the release of pro-inflammatory and pro-fibrotic cytokines, including interferon-gamma (IFN-gamma), chemokine C-C motif ligand 3 (CCL3) and chemokine C-C motif ligand 4 (CCL4) after migrating from the bloodstream to lung tissue in response to monocyte chemoattractant protein –1 stimulation [25].

Conversely, lung macrophages are classified into two distinct subsets: alveolar macrophages (AMs) and interstitial macrophages. AMs undergo a polarization process that drives their differentiation into classically activated macrophages (M1) or alternatively activated macrophages (M2). This polarization is influenced by the specific cytokine milieu [25,26]. While M1 polarization, driven by IL-4 and IL-13, induces pro-inflammatory status, M2 can exert anti-inflammatory and pro-fibrotic activity through TGF-β secretion [17,25,26]. Then, both abnormal activation and increased serum levels of B and T lymphocytes, as well as type-II ILCs, might drive pro-fibrotic activity by increasing the secretion of inflammatory cytokines such as IL-4, interleukin-5 (IL-5), interleukin-9 (IL-9), and IL-13 [25,26,27].

Hence, the recurrent injury of type-I alveolar epithelial cells (AECIs) represents the primary “primum movens” of IPF pathogenesis in genetically predisposed individuals [28,29]. All the previous mechanisms contribute to trigger the AECIIs dysfunction, which serves as the progenitors of AECIs, thereby perpetuating disease progression [6,7,30]. These processes induce EMT, leading to fibroblast activation, which is mediated by TGF-β and other pro-fibrotic pathways, ultimately resulting in ECM deposition and fibrotic degeneration of the lung interstitium [6,28,30]. In summation, the development of IPF is grounded in the interaction between genetic predisposition and exposure to environmental noxae such as smoking, infections and pollutants. These triggers, along with epigenetic modifications, cellular senescence, immune system imbalance as well as mitochondrial and proteostasis dysfunction impair the regenerative capacity of lung interstitium. This persistent injury of both AECIs and AECIs induces the EMT and activates fibroblasts, leading to an abnormal ECM deposition that results in a progressive fibrotic remodeling of the lung tissue.

## 4. Serum Biomarkers in Idiopathic Pulmonary Fibrosis

As of now, our understanding of IPF pathogenesis is continuing to evolve alongside the identification and integration of novel biomarkers. This process aims at enhancing the diagnostic accuracy and prognostic prediction beyond the traditional clinic, functional, and radiological approach. In the last few years, circulating biomarkers have emerged as intriguing and less invasive alternatives to assess and predict disease natural history. Amid these promising candidates, the quantity of matrix metalloproteinases-7 (MMP-7) was significantly higher in the plasma samples of IPF patients compared to controls, suggesting its potential prognostic role, particularly in combination with other markers [31]. Moreover, both Surfactant protein D (SP-D) and periostin were assessed in the serum of deceased and living IPF patients, respectively [32,33]. While SP-D concentrations were elevated and showed a negative correlation with DLco after 12 months of follow-up, increased periostin levels were associated with poorer overall survival in IPF individuals receiving nintedanib treatment [32,33]. In addition, Progranulin (PGRN) levels were significantly higher in non-IPF ILD compared to IPF or healthy controls, suggesting that it could play a role in differentiating these conditions, especially when radiological patterns are inconclusive. Although stable IPF patients may exhibit PGRN levels similar to healthy individuals, elevated PGRN concentrations may indicate an acute exacerbation [34].

The utility of biomarkers extends significantly into prognostication and monitoring disease progression. A panel of circulating biomarkers, including osteopontin (OPN), MMP-7, intercellular adhesion molecule-1 (ICAM-1), and periostin, were aggregated into a “progression index” that markedly enhances risk stratification for disease progression, mortality, and progression-free survival. This composite index might increase the predictive capacity of the clinical gender–age–physiology (GAP) index alone [35]. Thus, in a recent Greek cohort study, elevated serum levels of Krebs von Den Lungen-6 (KL-6), SP-D, VEGF-A, IGFBP-1, ICAM-1, and C-C motif ligand-18 (CCL18) were measured in sera samples of IPF individuals using ELISA. In particular, this study highlighted that the combination of elevated serum concentrations VEGF-A, KL-6, SP-D, and the GAP index may be associated with poor outcomes in IPF [32]. Among them, KL-6 is a glycoprotein secreted by injured AECIIs that has been recognized as a validated biomarker reflecting disease activity in ILDs [36,37]. Indeed, KL-6 serial measurements could be valuable to assess disease progression over time in ILDs, including IPF, while baseline serum levels may predict the risk of AE-IPF [37,38].

Recently, augmented concentrations in bronchoalveolar lavage fluid (BALF) and blood samples of S100A12, a protein primarily expressed by monocytes, were associated with increased mortality, shorter transplant-free survival, and reduced progression-free survival in IPF individuals. Notably, the combination of S100A12 with the GAP index may improve prognostic accuracy and offer a more comprehensive risk stratification tool [39]. Then, ECM remodeling, a hallmark of IPF, can be monitored through degradation products of ECM proteins like collagen, which are known as neoepitopes. In this context, elevated levels of collagen neoepitopes (e.g., C1M, C3M, C6M, PRO-C3, PRO-C6) correlate with increased disease severity, short-term progression, and long-term mortality [40]. Even routine complete blood count parameters such as elevated monocyte count have shown a correlation with worse clinical outcomes and more advanced disease at baseline. An increased monocytes count CD14+, assessed by flow-cytometry, has been found in IPF patients compared to controls as well as in individuals with a progressive phenotype of IPF compared to those with a non-progressive form [41]. Furthermore, a higher monocytes count has been associated with progression, risk of hospitalization, and mortality in IPF patients [42]. However, despite the promising role of these molecules, no serum biomarkers have been specifically approved in clinical practice for IPF management.

## 5. Neutrophils and Lymphocytes in Idiopathic Pulmonary Fibrosis

### 5.1. Neutrophils

**Figure 2:**
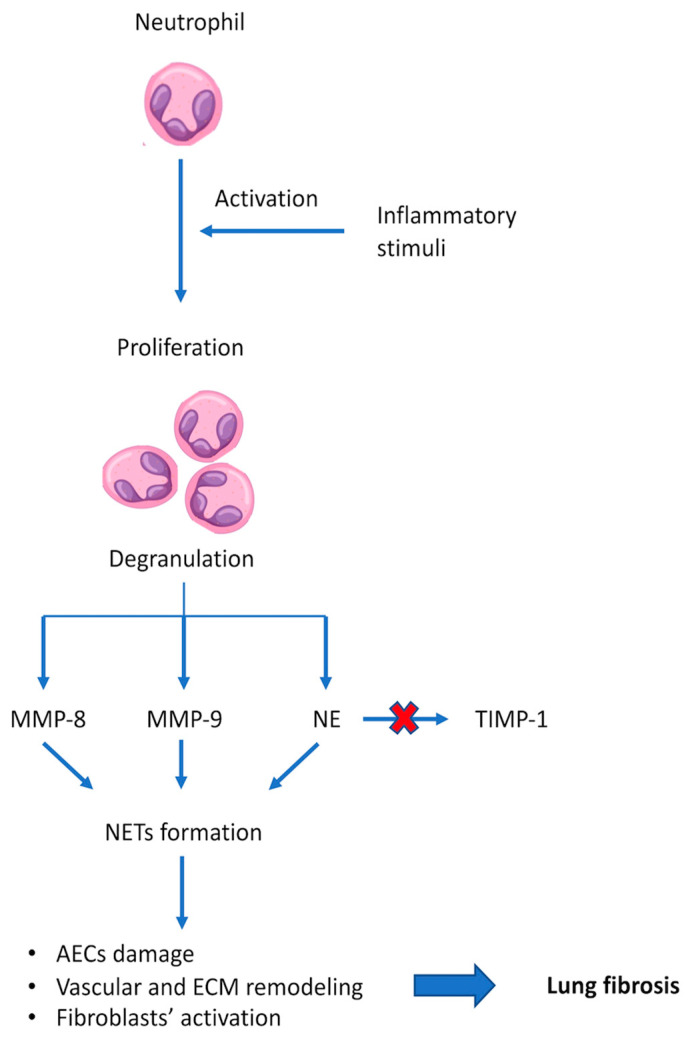
**The role of neutrophils in idiopathic pulmonary fibrosis development.** Neutrophils can be activated by inflammatory stimuli, leading to their proliferation and degranulation. This process leads to the release of MMP-8 and MMP-9, which directly promote NET formation, while NE can trigger NET after TIMP-1 inactivation. NETs can induce lung fibrosis development by triggering damage to alveolar epithelial cells, ECM, and vascular remodeling and fibroblast activation. **Abbreviations:** MMP-8—metalloproteinases-8; MMP-9—metalloproteinases-9; NE—neutrophil elastase; TIMP-1—tissue inhibitor of metalloproteinases-1; NET—neutrophils extracellular trap; AECs—alveolar epithelial cells; ECM—extracellular matrix.

Neutrophils are innate immune cells, produced in bone marrow, that form approximately 60% of the circulating white blood cells (WBCs) in humans [43]. Physiologically, these WBCs exert their antimicrobial effect via phagocytosis, degranulation, and, subsequently, the release of pro-inflammatory effectors such as cytokines, chemokines, protease, oxidase, and metalloproteinases (MMPs) [43,44]. Among them, MMP-8, MMP-9 and NE, a serine protease, are extracellular enzymes released through a priming-induced degranulation that can trigger extracellular NET formation [25,44,45]. Moreover, NE can inactivate tissue inhibitors of metalloproteinases-1 and, subsequently, induce NET formation [44,45]. This process, also known as NETosis, is a programmed cell death mechanism of neutrophils regulated by Gasdermin D (GSDMD) [34]. It is a pore-forming protein that triggers pyroptosis, a pro-inflammatory mechanism that leads to neutrophils death. Notably, upon cleavage by serine protease, GSDMD induces the secretion of neutrophils’ enzymes via degranulation, resulting in the NET formation process [46,47]. NETs are protein-based, interlinked structures, composed of decondensed chromatin and mitochondrial DNA, that inhibit microorganisms’ dissemination through the bloodstream [48]. In predisposed individuals, these enzymes may alter ECM composition, compromise AECs integrity, prime fibroblasts’ differentiation and induce lung vascular remodeling, thereby contributing to pulmonary fibrosis development [25,49]. According to their role in lung tissue remodeling, higher NETs levels in bronchoalveolar lavage fluid (BALF) of IPF patients were negatively correlated with lower forced vital capacity (FVC) (percent-predicted (%pp)), diffuse lung capacity for carbon monoxide (DLco) %pp and a worse 5-year prognosis [50]. Thus, increased NETs concentrations have been observed in bleomycin-induced mice models of fibrosis, suggesting that neutrophils may be overactivated and play a role in the development of pulmonary fibrosis [51]. Conversely, the inhibition of NE ameliorated lung fibrosis in bleomycin-induced mice by reducing collagen deposition [52]. In this context, while the MMP-9 concentration was found to be higher in sputum, NE-alpha-1-antitrypsin complexes and citrullinated histone 3, an indicator of NETs levels, was found to be increased in the BALF of IPF patients [53,54]. Both NE and myeloperoxidase (MPO), a neutrophil oxidase and marker of NET formation, were found to be elevated in pulmonary vessels of IPF tissue [55]. Similarly, MPO, citrullinated histones, and extracellular DNA have also been identified in fibrotic lung tissue as potential indicators of NET formation [56]. Hence, due to their pro-inflammatory and cytotoxic role, it may be hypothesized that NET function may mechanistically bridge the NLR with lung tissue damage, playing a role in lung fibrosis development (Figure 2). However, further research is warranted to establish this association and the underlying signaling pathways.

### 5.2. Lymphocytes

**Figure 3:**
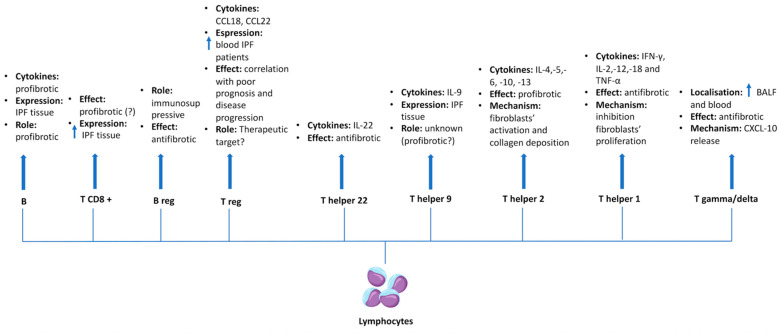
**The different roles of lymphocytes in idiopathic pulmonary fibrosis.** Lymphocytes subsets and their associated cytokines may exert distinct and sometimes opposing roles in IPF pathogenesis. Amid T cells, T-helper 1 (Th1) and T helper 2 (Th2) are known to have contrasting effects; Th1 cells exhibit an antifibrotic role by inhibiting fibroblasts’ proliferation and secreting cytokines such as IFN-gamma, IL-2, IL-12, IL-18, and TNF-alpha. In contrast, Th2 cells promote pro-fibrotic processes through the release of IL-4, IL-5, IL-6, IL-10, and IL-13 which trigger fibroblasts activation and collagen deposition. As per Th1 cells, T gamma/delta levels were found to be increased in BALF and peripheral blood of IPF patients. These cells may contribute to antifibrotic activity through the release of CXCL-10 as well as T helper 22, which exert antifibrotic effects via IL-22 secretion. In contrast, while B lymphocytes were found in IPF tissue and can secrete pro-fibrotic cytokines, the hypothetical pro-fibrotic roles of T CD8+ cells and Th9 cells remain controversial, despite the increased expression of T CD8+ cells in IPF tissue. Finally, Regulatory T and B cells (T reg and B reg) also displayed different effects. B reg appeared to have immunosuppressive and antifibrotic roles, whereas T reg was found to be increased in the peripheral blood of IPF patients, highlighting a correlation with poor prognosis and disease progression. **Abbreviations:** Th1—T-helper 1; Th2—T-helper 2; JFN-gamma—interferon-gamma; IL-2—interleukin-2; IL-12—interleukin-12; IL-18—interleukin-18; TNF-alpha—tumor necrosis factor-alpha; IL-4—interleukin-4; IL-5—interleukin-5; IL-6—interleukin-6; IL-10—interleukin-10; IL-13—interleukin-13; BALF—bronchoalveolar lavage fluid; CXCL-10—C-X-C motif chemokine ligand-10; IPF—idiopathic pulmonary fibrosis; T reg—regulatory T cells; B reg—regulatory B cells.

The role of lymphocytes in IPF is being recognized as complex and multifaceted due to the heterogeneity of lymphocyte subtypes and their involvement in a dysregulated immune environment which characterizes lung fibrosis [57]. These adaptive cells encompass a wide array of subtypes that might play different roles in pulmonary fibrosis [58]. Gamma delta T cells, which typically constitute from 1% to 10% of the total lymphocyte T population, were found to be increased in both the BALF and bloodstream of IPF patients, and may exert antifibrotic effects through the production of CXC chemokine ligand-10 [25,58,59]. Within lymphocytes T, the alternative polarization of T helper cells into the T helper 1 (Th1) or T helper 2 (Th2) subset is related to contrasting effects on fibrogenesis; Th1 cells inhibit fibroblasts’ proliferation and fibrotic degeneration through the secretion of IFN-gamma, IL-2, tumor necrosis factor-alpha, IL-12 and IL-18 [25,58,60]. In contrast, Th2 cells activate proliferation of fibroblasts and collagen deposition by releasing pro-fibrotic cytokines such as IL-4, IL-5, IL-6, IL-10, and IL-13 [25,58,60]. Additionally, while IL-9, produced by Th9 cells, and its receptors were found in higher quantities in human samples of IPF tissue, Th22 cells appeared to reduce fibrotic degeneration through the secretion of IL-22 in bleomycin mice models of pulmonary fibrosis [61,62]. Amid lymphocytes, regulatory T cells (T regs) are a subpopulation of T cells that exert immunoregulatory roles and might be related to fibrosis progression [25]. In such a scenario, single-cell RNA sequencing of peripheral blood from IPF patients has shown a reduction in total lymphocyte counts, particularly in those with progressive disease, followed by higher levels of circulating T regs [63]. This augmented serum concentration of T regs and their chemokines, such as CCL18 and CCL22, may be associated with poorer survival and could correlate with IPF severity, suggesting a potential therapeutic target [26,63,64]. Concerning other immune cell subtypes, single-cell transcriptomics of lung tissue has indicated that there is an increased percentage of CD8+ T cells in IPF patients, exhibiting altered metabolic and signaling pathways associated with fibrosis, suggesting their potential pro-fibrotic role [65]. In contrast, regulatory B cells, a subset of B lymphocytes with immunosuppressive functions, might inhibit Th cells playing an antifibrotic role in IPF [26,66]. As per T lymphocytes, an abnormal activation of B lymphocytes, along with higher concentrations of B cells and their cytokines, have been observed in both in bleomycin mice models of pulmonary fibrosis and IPF tissue [25] (Figure 3). However, all these preliminary studies suggest that the roles of different lymphocyte subsets and their secreted molecules in fibrogenesis are still poorly understood.

## 6. Neutrophil-to-Lymphocyte Ratio (NLR)

**Figure 4:**
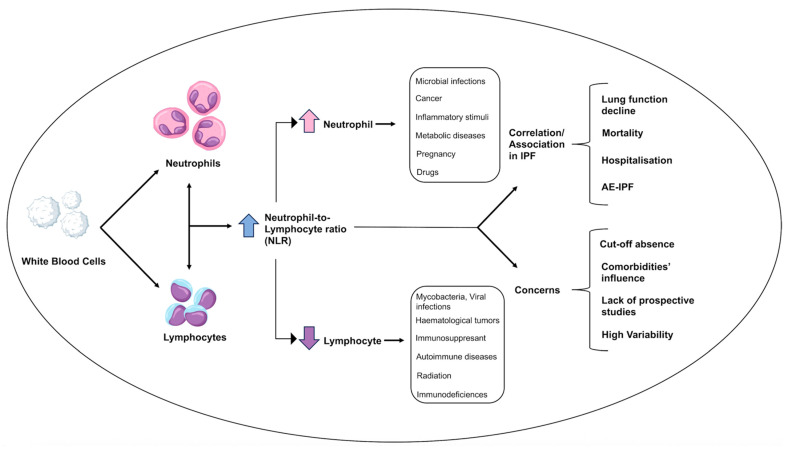
**Variability, limits and potential application of the neutrophil-to-lymphocyte ratio in idiopathic pulmonary fibrosis:** Neutrophil-to-lymphocyte ratio (NLR) values can increase due to a wide array of factors that can augment the blood neutrophil count or decrease circulating lymphocyte count. While microbial infection, cancer, inflammatory stimuli, metabolic diseases, stress conditions, and drugs (e.g., corticosteroids, noradrenaline, lithium, granulocyte–macrophage colony-stimulating factor) can trigger an increase in neutrophil counts, a decrease in circulating lymphocytes can be induced by Mycobacteria infections (e.g., Mycobacterium tuberculosis, non-tuberculous mycobacteria), viral infections (e.g., Epstein–Barr, HIV and hepatitis B), autoimmune diseases (e.g., Sjogren syndrome, rheumatoid arthritis, systemic lupus erythematosus), hematological malignancies (e.g., non-Hodgkin lymphoma, myelodysplastic syndrome), and drugs (e.g., glucocorticoids, chemotherapy and immunosuppressants), radiation, and primary immunodeficiencies. Although NLR values have been linked to lung function decline, acute exacerbation of idiopathic pulmonary fibrosis, mortality, and increased risk of hospitalization, their clinical utility is limited due to the lack of cut-off thresholds and prospective studies, the influence of comorbidities, and high variability. **Abbreviations:** NLR—neutrophil-to-lymphocyte ratio; IPF—idiopathic pulmonary fibrosis; AE-IPF—acute exacerbation of idiopathic pulmonary fibrosis.

NLR is an inexpensive, readily available, and clinically applicable biomarker of systemic inflammation which reflects the interplay between innate and adaptive immunity [67]. Since the COVID-19 pandemic, NLR has gained attention as a biomarker of inflammation and a prognostic indicator across various conditions such as sepsis, atherosclerosis, chronic kidney disease, chronic obstructive pulmonary disease (COPD), tumors, and other diseases [3,4]. In both COVID-19 infection and sepsis, higher NLR values have been recognized as a predictor of clinical outcomes and prognosis [68,69]. As per these conditions, increased NLR values were detected in the blood of COPD patients, both in stable disease or in the course of AE-COPD, compared to the blood of healthy individuals [70]. Similarly, due to its association with atherosclerotic events, elevated NLR values may represent a useful tool for risk stratification even in the presence of a normal WBC count, and may be a predictor of mortality for all causes and cardiovascular events in chronic kidney disease patients and poor clinical outcomes in cancer patients [71,72,73]. Although it has been recognized as a highly sensitive marker that can rapidly fluctuate under inflammatory processes, its specificity remains limited, as it can be augmented by various non-specific stimuli [74]. Among these, numerous triggers can increase the peripheral blood neutrophil count or decrease the lymphocytes count, ultimately leading to an elevated NLR [75,76]. An increase in the neutrophil blood count, also known as “neutrophilia”, is one of the most common laboratory findings in clinical practice [75]. This condition can be triggered by a variety of mechanisms such as pathological causes (i.e., microbial infections, metabolic diseases, cancer) and paraphysiologic factors (i.e., pregnancy, stress factors) [74,75]. In addition, several drugs such as corticosteroids, noradrenaline, lithium, and the administration of granulocyte–macrophage colony-stimulating factor may raise the circulating neutrophil count [74]. Conversely, lymphocytopenia is characterized by a diminishing peripheral blood lymphocytes count, which in turn can be provoked by several processes [76]. These include infections (i.e., Mycobacterium tuberculosis, non-tuberculous mycobacteria, Epstein–Barr virus, hepatitis B infection, and HIV), autoimmune disorders (e.g., Sjogren syndrome, rheumatoid arthritis, and systemic lupus erythematosus) and malignancies such as non-Hodgkin lymphoma and myelodysplastic syndrome. Less common causes include glucocorticoids, chemotherapy and immunosuppressants, primary immunodeficiencies, and radiation [74,76] (Figure 4).

### 6.1. Setting the Bar: Defining the Optimal Neutrophil-to-Lymphocyte Ratio Threshold

Although several studies suggested that there may be an association between increased NLR and higher mortality or poorer clinical outcomes, its values can widely differ within the population [77,78]. In the last few years, several studies have been conducted to identify the optimal NLR value within the population. Notably, demographics factors (sex, race, age and body mass index), smoking, environmental exposures, and comorbidities may influence NLR [79]. Both Hispanic and Non-Hispanic Black individuals exhibited lower average NLR values compared to Non-Hispanic White subjects [79]. Moreover, data from other international cohorts such as Korean, Chinese, and Iranian populations have reported mean NLR values that range from 1.5 to 2.0 both in men and women [80,81,82,83]. Similarly, a wider range of NLR values, between 0.78 and 3.53, have been proposed as physiologic in healthy individuals [4]. The influence of demographic factors in NLR variation was also confirmed by the finding that its values may tend to increase with age [84]. Finally, three different ranges of NLR values have been proposed to distinguish between physiological and pathological conditions. Values between 1.0 and 2.0 were considered as physiological, whereas a ratio > 3 was indicative of underlying pathological condition. Amid these, a “gray zone” (NLR 2.3–3.0) has been identified as potentially associated with an increased risk of pathological conditions such as inflammatory processes, cancer, and cardiovascular or infectious diseases [74] (Table 1). However, a physiological NLR value has yet to be established due to the high variability across the population, the clinical context, and underlying conditions of this inflammatory marker, which limit its standardization.

### 6.2. The Role of the Neutrophil-to-Lymphocyte Ratio in Idiopathic Pulmonary Fibrosis

In IPF, the lack of validated biomarkers still represents a major limitation which hinders advances in disease management and therapeutic approach. Two antifibrotic drugs, pirfenidone and nintedanib, have been approved for IPF treatment: pirfenidone is a pyridine with pleiotropic properties that provides antifibrotic, anti-inflammatory, and anti-oxidant effects, while nintedanib is a tyrosine-kinase inhibitor which interferes with the progression of lung fibrosis [29,85,86,87]. Overall survival remains variable and is often influenced by concomitant comorbidities including pulmonary hypertension, gastroesophageal reflux, obstructive sleep apnea, lung cancer and emphysema [87,88]. Therefore, IPF current monitoring is one of the key strategies of disease management and is based on the assessment of lung volumes (FVC) and gas exchange (DLco), respiratory symptoms worsening, and radiological alterations on high-resolution computed tomography [30]. In addition, the FVC is considered as the most consistent functional parameter, and a decrease from 2% to 6% is associated with a negative impact on disease progression [89]. To date, several studies have assessed the prognostic role of NLR in IPF, including its association with clinical outcomes, treatment response and risk of AE-IPF.

### 6.3. Neutrophil-to-Lymphocyte Ratio in Idiopathic Pulmonary Fibrosis Monitoring

Amid blood biomarkers, several studies assessed the role of NLR as a biomarker to predict clinical outcomes and monitor IPF course. Firstly, Nathan et al. conducted a post hoc analysis on two phase III double-blind randomized controlled trials (RCTs) named ASCEND and CAPACITY [9,90,91]. These RCTs included 1334 IPF patients, aged from 40 to 80, who underwent antifibrotic treatment with pirfenidone or placebo administration [90,91]. The analysis indicated that significant changes in the NLR over a period of 12 months may predict worsening respiratory symptoms, lung function decline, increased risk of hospitalization, and mortality [9].

These findings were also supported by Achaiah et al., who performed a retrospective study on 128 patients affected by IPF. A statistically significant association between NLR and FVC decline was found [92]. Furthermore, the association between NLR and lung function decline was also assessed in BALF samples from IPF individuals. Accordingly, both FVC and forced expiratory volume in 1 s were inversely correlated with NLR [93]. These studies suggest that NLR may have potential as a monitoring tool in IPF, due to its correlation with clinical outcomes and lung function declines. Nevertheless, all these preliminary findings need to be validated in larger studies.

### 6.4. Neutrophil-to-Lymphocyte Ratio as Prognostic Parameter in Idiopathic Pulmonary Fibrosis

Historically, IPF has a poor prognosis, with median survival ranging from 3 to 5 years from diagnosis in the absence of antifibrotic treatment [6,7]. Despite the approval of antifibrotic drugs that slow down functional decline and disease progression, its clinical course still remains highly unpredictable [1,6,7]. Several studies were conducted to assess the prognostic role of NLR in IPF individuals. Within these studies, NLR values greater than 2.77 were significantly associated with poorer prognosis in IPF patients [92].

Another crucial observational study by Mikolasch et al. underscored the potential prognostic utility of NLR in IPF. Among 999 IPF patients, an NLR > 2.9 was associated with worse survival compared to lower values. The potential prognostic role of NLR was also enforced by its strong association with the GAP index and GAP stage, a validated prognostic index used to assess disease severity and predict mortality in IPF [94].

In addition, Chen et al. calculated NLR values in a cohort of IPF individuals and showed that NLR had a negative correlation with PaO_2_/FiO_2_ ratio and its higher value could predict reduced overall survival [95]. Despite the absence of validated cut-off values both in healthy people and in patients with IPF, these findings suggest that baseline NLR values may be higher in IPF individuals compared to healthy controls, and that their variation over time could offer prognostic insights and could be useful for risk stratification. Nevertheless, these intriguing hypotheses need to be confirmed in larger studies.

### 6.5. Neutrophil-to-Lymphocyte Ratio in Acute Exacerbation of Idiopathic Pulmonary Fibrosis

AE-IPF is a severe and life-threatening event of uncertain origin, typically occurring in individuals with advanced disease, that is related to rapid disease progression and high mortality [96]. Among AE-IPF patients, NLR on day 1 may serve as a predictor of survival at 90 days, while NLR values on day 4 and day 8 appeared to be useful for monitoring the need for oxygen and severity of respiratory failure [11].

### 6.6. Neutrophil-to-Lymphocyte Ratio and Antifibrotic Treatment

To date, only a few studies have been conducted regarding the role of NLR in IPF patients who received antifibrotic therapy. In such a scenario, higher NLR value has been associated with an increased risk of mortality, while changes in NLR within 12 months may be linked to poor clinical outcomes such as lung function decline, augmented risk of mortality and hospitalization, and poor quality of life, despite treatment with antifibrotic drugs (Table 2) [9,10].

## 7. Conclusions and Future Perspectives

Recent studies suggest that NLR is a cheap and minimally invasive parameter that has been associated with mortality, lung function decline, acute exacerbation, and risk of hospitalization in IPF patients. These findings suggest that NLR might be used as a potential non-invasive tool for risk stratification and disease monitoring. Moreover, according to neutrophils and lymphocytes’ role in IPF pathobiology, the NLR reflects the inflammatory status, driven by the interplay between the immune and adaptive system, which represents a key feature of IPF pathogenesis. However, several concerns can reduce its reliability in clinical practice. Firstly, a lack of specificity and the absence of validated cut-off values that reliably distinguish physiological and pathological conditions, as well as its variability in the presence of comorbidities, represent significant limitation for its application in clinical practice. Several factors, such as immunosuppressive drugs, antibiotics, stress, and dietary supplements could induce a variation in neutrophil and lymphocyte counts, potentially leading to a dysregulation of the signal that controls immune cells trafficking, including the release of neutrophils and lymphocytes into the bloodstream and their localization into inflamed or damaged tissues. To move beyond summary and into suggestion, NLR may be integrated with other clinical parameters such as FVC and DLco into a composite risk score to improve prognostic stratification. Then, NLR could be added as a secondary endpoint in future pilot studies to assess its role as a biomarker of disease progression and antifibrotic treatment response. All in all, these preliminary findings suggest that NLR may be a promising biomarker in IPF management that needs to be studied in larger prospective studies.

## Figures and Tables

**Table 1:** Summary of the studies assessing neutrophil-to-lymphocyte ratio cut-off values.

Study	Proposed NLR Cut-Off	Population/Conditions	References
Azab et al. (2014)	1.76 2.08 2.24	Non-Hispanic Black Hispanic Non-Hispanic White	[79]
Kweon et al. (2016)	1.53 1.54	Healthy Korean women Healthy Korean men	[83]
Forget et al. (2017)	0.78–3.53	Healthy South Korean subjects	[4]
Lee et al. (2018)	1.65 ± 1.96 (mean ± SD)	Healthy South Korean subjects	[80]
Fest et al. (2018)	1.88 1.68 1.63 2.13 1.76	Males Females Individuals aged from 45 to 54 y/o Individuals older than 85 y/o Average values in analyzed sample	[84]
Moosazadeh et al. (2019)	1.70 ± 0.70 (mean ± SD)	Healthy Iranian people	[82]
Wu et al. (2019)	1.59 ± 0.59 (mean ± SD) 1.62 ± 0.64 (mean ± SD)	Healthy Chinese men Healthy Chinese women	[81]
Zahorec et al. (2021)	1.0–2.0 2.3–3.0 >3.0	Physiological range “Gray zone” Pathological values	[74]

**Table 2:** Summary of the studies pertaining to the neutrophil-to-lymphocyte ratio in patients with idiopathic pulmonary fibrosis.

Study	Outcomes	References
Achaiah et al.	Association between NLR and lung function decline Association between NLR values > 2.77 and poor prognosis	[92]
D’alessandro et al.	Inverse correlation between NLR and lung function parameters (FVC and forced expiratory volume at 1 s)	[93]
Nathan et al.	Significant changes in NLR over 12 months may be linked to an increased risk of worsening respiratory symptoms, lung function decline, hospitalization, and mortality in IPF patients undergoing antifibrotic treatment	[9]
Mikolasch et al.	Association between NLR value > 2.9 and poorer survival compared to lower values NLR association with GAP stage and GAP index	[94]
Chen et al.	Negative correlation between NLR value and PaO_2_/FiO_2_ ratio Higher NLR value could predict reduced overall survival	[95]
Arai et al.	NLR value at 1 day may predict survival at 90 days in AE-IPF NLR values on day 4 and 8 may predict the need for oxygen and severity of respiratory failure	[11]
Takuma et al.	Association between higher NLR value and increased risk of mortality in patients treated with antifibrotic therapy	[10]

## Data Availability

No new data were created or analyzed in this study.

## Abbreviations

The following abbreviations are used in this manuscript:

NLR	neutrophil to lymphocyte ratio
IPF	idiopathic pulmonary fibrosis
ILDs	interstitial lung diseases
f-ILD	fibrotic interstitial lung disease
UIP	usual interstitial pneumonia
AE-IPF	acute exacerbation of idiopathic pulmonary fibrosis
MiRNA	microRNA
EMT	epithelial-myofibroblasts’ transition
TGF-β	transforming growth factor-beta
AECIIs	Type-II alveolar epithelial cells
ILCs	immune lymphoid cells
ECM	extracellular matrix
NE	neutrophil elastase
NET	neutrophil extracellular trap
IL-4	interlukin-4
IL-10	interleukin-10
IL-13	Interleukin-13
IFN-gamma	Interferon-gamma
CCL3	chemokine C-C motif ligand 3
CCL4	chemokine C-C motif ligand 4
AMs	alveolar macrophages
M1	classically activated macrophages
M2	alternatively activated macrophages
IL-5	interleukin-5
IL-9	interleukin-9
AECIs	type-I alveolar epithelial cells
WBCs	white blood cells
MMPs	metalloproteinases
MMP-9	metalloproteinase-9
MMP-7	matrix metalloproteinases-7
AECs	alveolar epithelial cells
MMP-8	metalloproteinase-8
BALF	bronchoalveolar lavage fluid
MPO	myeloperoxidase
Th1	T helper 1
Th2	T helper 2
T regs	regulatory T cells
FVC	forced vital capacity
DLco	diffuse capacity for carbon monoxide
RCTs	randomized controlled trials
%pp	percent predicted
GSDMD	Gasdermin D
SP-D	Surfactant protein D
OPN	Osteopontin
GAP index	Gender-age-physiology index
ICAM-1	intercellular adhesion molecule-1
KL-6	Krebs von Den Lungen-6
CCL18	C-C motif ligand-18
COPD	Chronic obstructive pulmonary diseases
